# Evaluating historical changes in a mussel bed community in northern California

**DOI:** 10.1038/s41598-025-86105-9

**Published:** 2025-01-14

**Authors:** Emily K. Longman, Sarah Merolla, Stefan A. Talke, Nicholas Trautman, John L. Largier, Leslie Harris, Eric Sanford

**Affiliations:** 1 Bodega Marine Laboratory, University of California, Davis, Bodega Bay, CA 94923 USA; 2 Department of Evolution and Ecology, University of California, Davis, Davis, CA 95616 USA; 3 The Ecosystems Center, Marine Biological Laboratory, Woods Hole, MA 02543 USA; 4 Department of Civil and Environmental Engineering, California Polytechnic State University, San Luis Obispo, CA 93407 USA; 5 Department of Environmental Science and Policy, University of California, Davis, Davis, CA 95616 USA; 6 Natural History Museum of Los Angeles County, Los Angeles, CA 90007 USA; 7 Department of Biology, University of Vermont, Burlington, VT 05405 USA

**Keywords:** Historical ecology, Foundation species, Global change, Climate-change ecology, Marine biology

## Abstract

Marine foundation species are increasingly impacted by anthropogenic stressors, driving a loss of diversity within these critical habitats. Prior studies suggest that species diversity within mussel beds has declined precipitously in southern California, USA, but it is unclear whether a similar loss has occurred farther north. Here, we resurvey a mussel bed community in northern California first sampled in 1941 to evaluate changes in diversity after 78 years. More broadly, we explore the value and potential challenges of using imperfect historical data to assess community changes. Our 2019 survey documented 90 species/taxa across 10 phyla. The majority of species (~ 72%) were common to all replicate plots, suggesting that variation in species diversity over small spatial scales was unlikely to mask temporal changes. In contrast to results from southern California, we observed no decline in species diversity between timepoints. However, there were shifts in species composition, with an increase in the abundance of southern species and a decrease in northern species, consistent with warming observed at a nearby shoreline site. Overall, our findings are an encouraging sign for the health of this mussel bed community in northern California and illustrate how non-traditional data can contribute to assessments of long-term ecological change.

## Introduction

Humans have negatively impacted global biodiversity through changes in land/sea use, pollution, invasive species, extraction of natural resources, and climate change^1–3^. Impacts of this biodiversity crisis include species extinctions, a reduction in population sizes and genetic diversity, and the loss of ecologically important functional traits^4,5^. Changes often cascade through the ecosystem, affecting functioning and productivity^6^ and subsequently impacting ecosystem services^2^.

Anthropogenic effects on natural communities are difficult to evaluate without quantitative baseline measurements. However, most ecological data sets typically span only a few years^7^, and historical sources of information are often scarce, underutilized, or forgotten. Recovering and evaluating archival records can help bridge temporal gaps between paleobiological and modern data sets and may provide new insights into ecological change^8–10^. These resources can take a variety of forms including unpublished reports, museum collections, maps or photographs, species lists from field surveys, traditional ecological knowledge, or other information not published in the scientific literature^11–13^. However, because many of these documents were collected without modern scientific goals and methods, there are often limitations. For example, records are typically fragmented, surveys may have minimal or no replication, and sampling methods may be poorly described or unknown, among other biases^9,14^. This raises questions regarding how researchers can best address the limitations of historical data sets to make them more informative for evaluating ecosystem change^12^.

Researchers in terrestrial systems have a long history of utilizing historical data^14–16^. For example, museum records and data sets spanning decades to centuries have been used to document phenological changes and range shifts in many plant and animal species in response to a changing climate^17–20^. In contrast, the use of historical resources in marine ecosystems is less common [but see^21–24^].

Marine foundation species, including corals, seagrasses, kelp forests and mussel beds, are often critical to community organization and biodiversity because they create a habitat matrix for many organisms to live within^25,26^. Many of these foundation species are threatened by human impacts and their abundance and associated ecosystem services are declining at a rapid rate^27–30^. In many temperate regions, mussels are important foundation species that create three-dimensional beds that serve as a structurally complex habitat matrix. Mussel beds provide a refuge from environmental stressors^31,32^ and predators^33^ and thus harbor a diverse assemblage of taxa^34,35^. These habitats are threatened by multiple anthropogenic stressors including climate change, pollution, harvesting, trampling, dredging, and trawling^29,36,37^.

The California mussel (*Mytilus californianus*) forms extensive mid-intertidal beds in the northeast Pacific that support a rich fauna of hundreds of species^38,39^. Previous research in southern California, USA, has documented declines in the area and three-dimensional structure of mussel beds^40^, and comparisons between historical data (1960s to 1970s) and more recent surveys have shown striking declines in the species richness of mussel bed inhabitants^41^. However, there is little information regarding whether similar declines in mussel bed diversity have occurred farther north. In addition to declines in species diversity in mussel beds and other intertidal habitats in California^42^, there have been climate-related shifts in the species composition of intertidal communities. In particular, the abundance of warm-adapted species with primarily southern geographic ranges has increased in California in association with warming temperatures^43–45^.

**Fig. 1:**
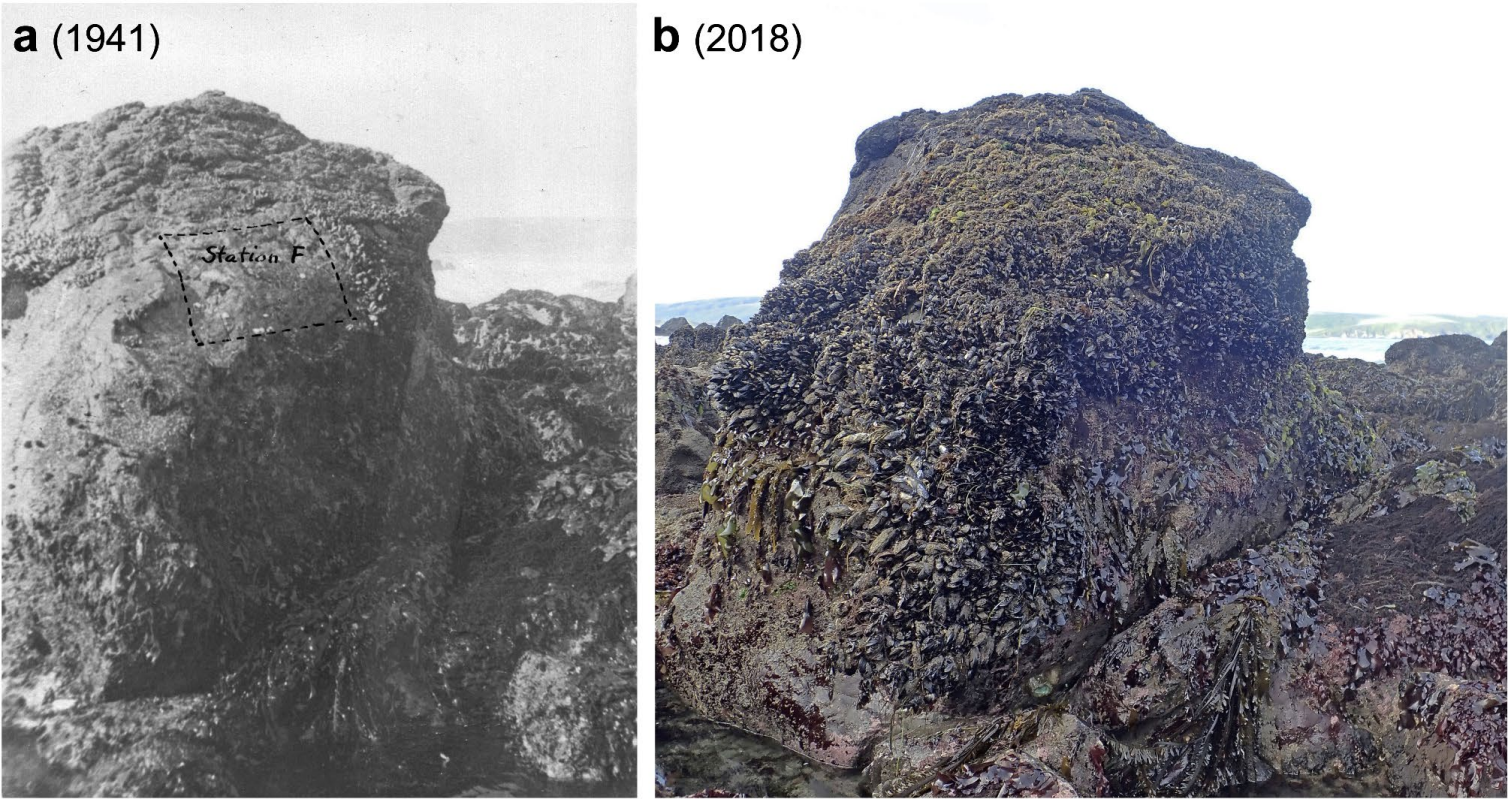
(**a**) Mussel bed plot (‘Station F’) on a large rock boulder at Dillon Beach, California, after sampling by graduate students Harvey I. Fisher and Milton Hildebrand in May/June 1941^46^. Dotted line outlines the area sampled for taxa living in the mussel bed (2.5 ft wide x 3 ft tall = 0.70 m^2^). (**b**) The same rock boulder photographed 77 years later in May 2018, about one year before resampling was conducted.

In this study, we resurveyed an intertidal mussel bed community near Dillon Beach in northern California that was first studied in 1941 as part of a field course taught at the University of California, Berkeley^46^. The researchers sampled a single mussel bed plot using a very large quadrat (area = 0.70 m^2^). They identified and counted thousands of organisms in the plot and measured the size structure of the mussel bed. Using photos and hand drawn maps from the report, we relocated and resampled the exact same mussel bed plot in 2019 (Fig. 1). The lack of plot replication in the original 1941 survey was a concern, so in 2019, we sampled four replicate mussel bed plots (quadrat area = 0.16 m^2^) within the same rocky point to evaluate whether local plot-to-plot variation was large enough to overwhelm detection of any temporal patterns of change. We hypothesized that this historical comparison of surveys would reveal an overall decline in species diversity and an increase in the relative abundance of warm-adapted species. These comparisons of survey data also serve as a case study regarding the potential value and challenges of using historical records to evaluate changes in natural communities. In particular, we explored whether the limited historical data available can inform an understanding of temporal changes within a mussel bed community given the potential for spatial variation within the habitat. Finally, we investigated whether ecological changes in this intertidal mussel bed community are consistent with observed changes in water temperature and exposure to aerial conditions (emersion times) over the ~ 80-year time period between surveys.

## Results

Across the plots sampled in 2019, we counted and identified over 37,000 individuals comprising 90 species/taxa across 10 phyla (Appendix S1). An estimated 45,573 individuals were found in the mussel plot in 1941, compared to 34,340 in our 2019 survey. The decline in total abundance was driven by a decrease in estimated acorn barnacle abundance from 37,000 (1941) to just under 18,000 (2019).

### Mussel size distribution

**Fig. 2:**
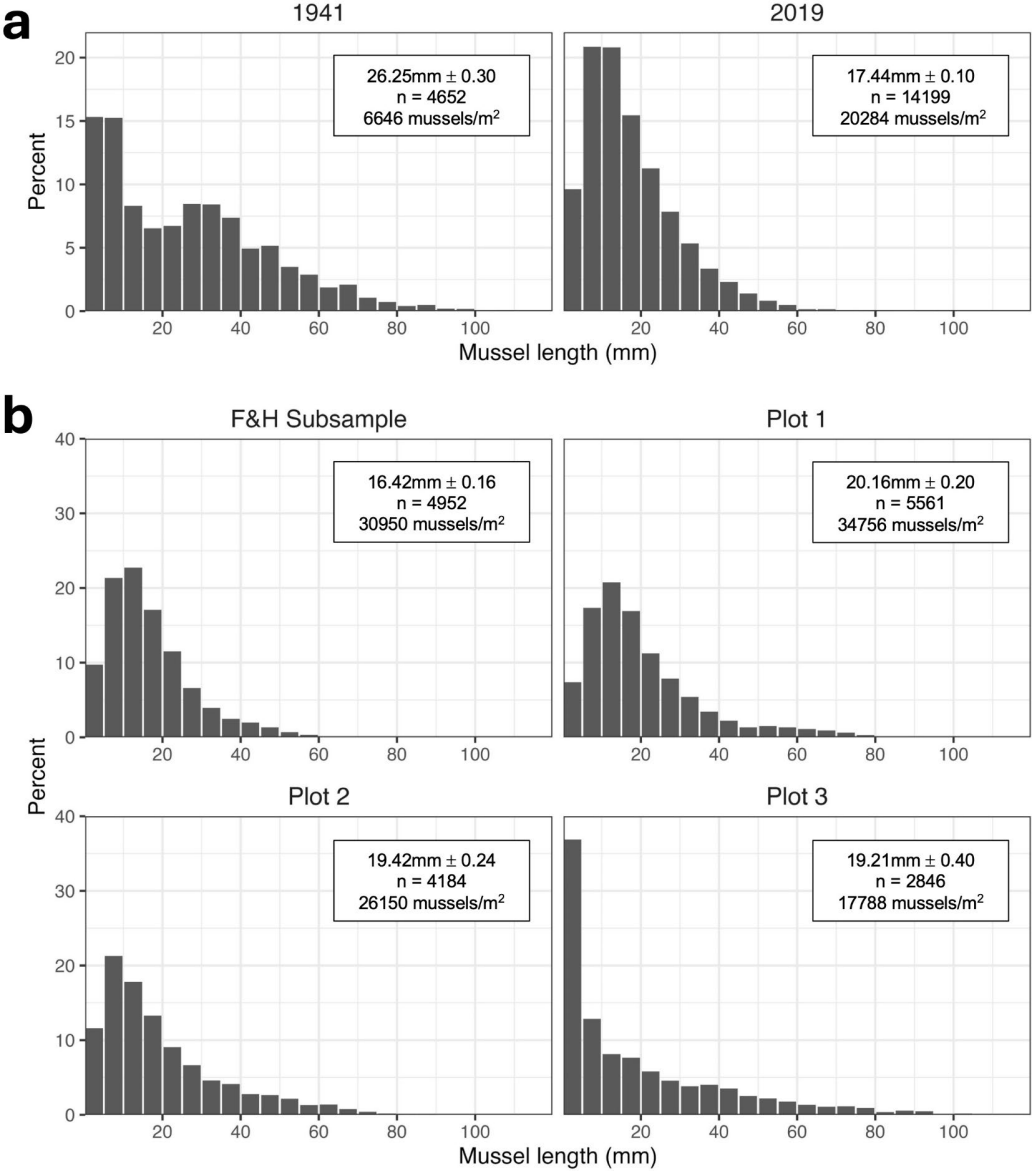
Size frequency distribution of mussels, *Mytilus californianus*, in field surveys. Bars are the percent of total mussels in each size category, grouped by 5 mm size bins. (**a**) Comparison of the historic survey in 1941 to the 2019 survey of the same large 0.70 m^2^ plot. (**b**) Comparison of the four 0.16 m^2^ replicate plots in 2019. ‘F&H Subsample’ is a subsample of the plot used in the temporal comparison. Mean mussel length ± standard error, sample size (n) and density (number of mussels/m^2^) are reported for each plot.

The 2019 resurvey of the original plot contained three times more mussels than the 1941 survey (14,199 vs. 4,652 mussels; Appendix S2: Table S2). The mean mussel length decreased slightly between 1941 and 2019, from 26.3 to 17.4 mm (Fig. 2a; W = 26211681, *p* < 0.001). The 1941 survey contained more medium to large mussels compared to 2019, and both surveys were dominated by small mussels (< 25 mm) (Fig. 2a; Chi-square test, χ^2^ = 2329.5, *df* = 21, *p* < 0.001).

Mussel size distribution was compared among four spatial replicates with one of the replicates (“F&H Subsample”) being a subsample of the original large plot sampled in 1941. The number of mussels in the four spatial replicates sampled in 2019 ranged from 17,788 to 34,756 mussels/m^2^ (Appendix S2: Table S2). Mean mussel bed depth also varied among the plots (Appendix S2: Fig. S1; ANOVA, F_3,48_ = 3.05, *p* = 0.038), and ranged from 4.5 to 17 cm. The mussel size distribution differed somewhat among the four replicates (Fig. 2b; Chi-square test, χ^2^ = 2449.1, *df* = 69, *p* < 0.001). In particular, Plot 3 had many very small mussels (less than 5 mm), whereas the other three plots were dominated by mussels 5–30 mm in length. Mean mussel length varied among plots, but within a relatively narrow range from 16.4 to 20.2 mm (Fig. 2b; Kruskal-Wallis test, χ^2^ = 278.96, *df* = 3, *p* < 0.001).

## Spatial variation in species diversity among replicate plots

**Fig. 3:**
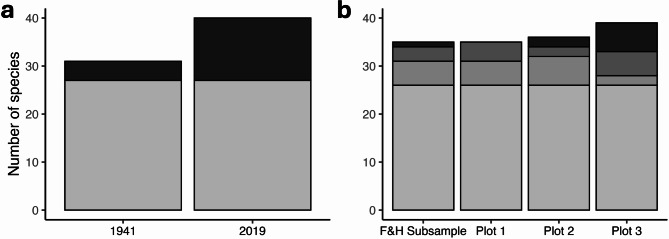
Differences in species richness in mussel bed communities for (**a**) the original large plot (0.70 m^2^) sampled in 1941 and 2019, and (**b**) a spatial comparison among four replicate plots (0.16 m^2^) sampled in 2019. The lightest grey color represents species present in both 1941 and 2019 (for a), or in all four of the plots (for b). Darker colors represent species present in only three or two plots (for b) and the darkest color represents species unique to that plot.

**Fig. 4:**
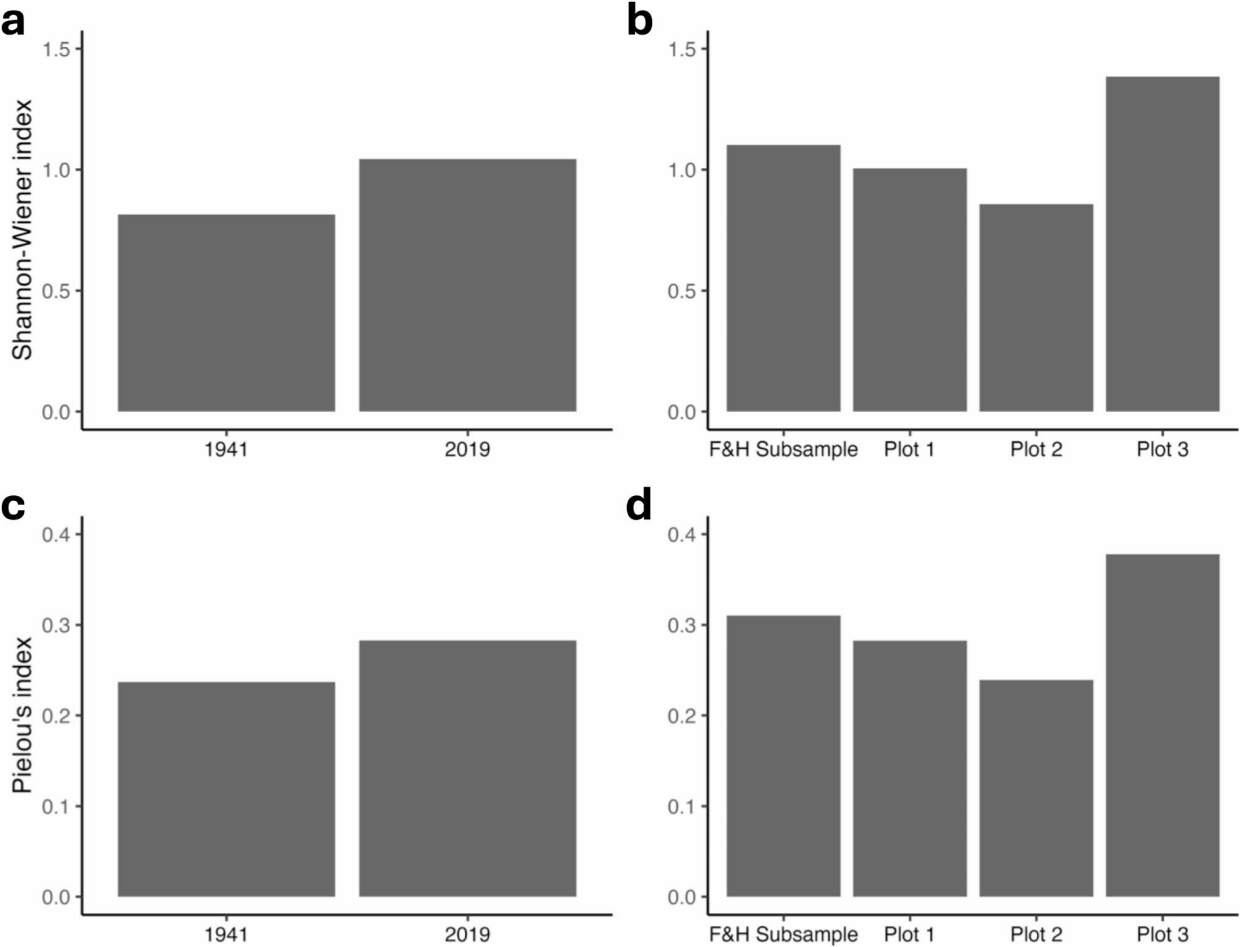
Species diversity in mussel bed plots as measured by the Shannon-Wiener index in (**a**) the temporal comparison, and (**b**) the spatial comparison. Species evenness as measured by Pielou’s Index for (**c**) the temporal comparison, and (d) the spatial comparison.

In 2019, species richness was generally consistent among the four spatial replicate mussel bed plots and ranged from 35 to 39 species, with 26 species common to all four replicate plots (Fig. 3b). One plot contained 9 unique species; 8 of these species were uncommon, with fewer than 5 individuals. Species diversity and evenness were also similar among three of the four replicates (Fig. 4b and d); Plot 3 stood out as having higher species diversity and evenness.

## Historical changes in species diversity

There was no evidence that species richness declined in the mussel bed plot between 1941 (31 species) and 2019 (40 species) (Fig. 3a). There were 27 species common to both the historical survey and the 2019 survey (Fig. 3a). Four species were unique to the 1941 survey and 13 were unique in the 2019 survey. Three of the four species that were present in 1941 but absent when the original plot was resurveyed were present in the other replicate plots. Only one species that was present in 1941 (the sea cucumber *Cucumaria pseudocurata*, with an abundance of 79 individuals) was not found in any of the 2019 replicate plots. Many of the species unique to the 2019 survey were very uncommon, with 10 of the 13 species numbering 10 or fewer individuals across all of the 2019 surveys. Species diversity and species evenness were greater in 2019 than in 1941 (Fig. 4a and c).

## Shifts in abundance

**Fig. 5:**
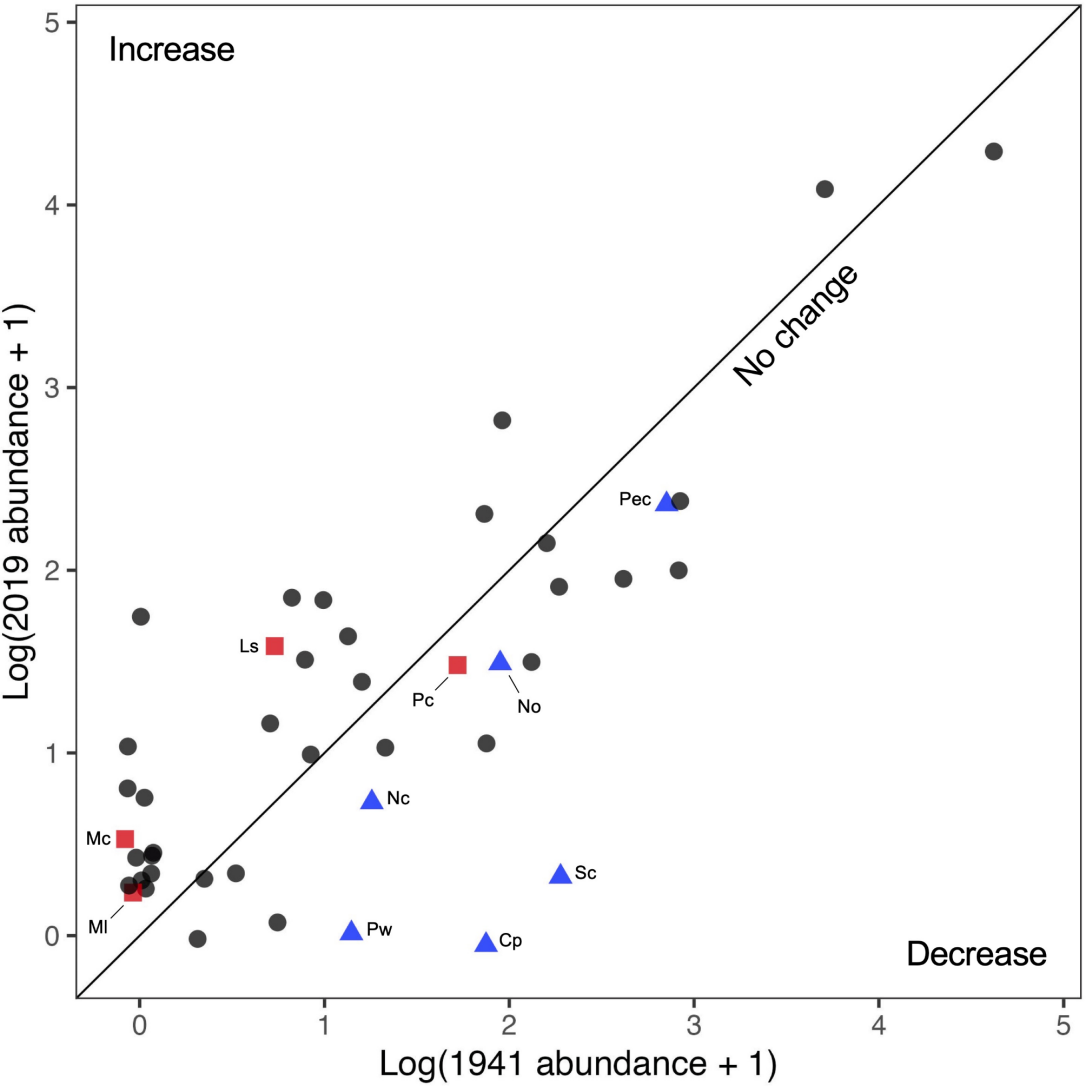
Changes in the log abundance of species within the mussel bed plot at Dillon Beach in 1941 versus 2019. Species are colored by their geographic range with southern, northern, and cosmopolitan species depicted in red squares, blue triangles, and dark grey circles, respectively (see Methods for classification of ranges). Points are slightly jittered to be able to see overlapping species. Species above the one-to-one line increased in abundance and species below the line decreased in abundance. Southern and northern species are labeled: *Phragmatopoma californica* (Pc), *Lottia scabra* (Ls), *Modiolus carpenteri* (Mc), *Mopalia lionata* (Ml), *Petrolisthes cinctipes* (Pec), *Semibalanus cariosus* (Sc), *Nucella ostrina* (No), *Cucumaria pseudocurata* (Cp), *Nucella canaliculata* (Nc), *Pentidotea wosnesenskii* (Pw).

Species abundances and densities varied greatly between the surveys in 1941 and 2019 (Table 1; Appendix S2: Table S3). No obvious trends occurred across larger taxonomic groupings; i.e., no phyla or classes showed consistent changes (Appendix S2: Fig. S2). However, community composition showed a trend consistent with the predicted effects of increased water temperatures. All six of the northern species decreased in abundance by 64–100%, and three of the four southern species increased in abundance (Fig. 5). Two of the northern species (the isopod *Pentidotea wosnesenskii* and the sea cucumber *Cucumaria pseudocurata*) were absent in the 2019 survey of the historical mussel bed plot. However, a few *P. wosnesenskii* individuals were found in the replicate plots, indicating that this species was still present at Dillon Beach (Table 1). In contrast, the sea cucumber was not identified in any of our surveys; this species disappeared from the region following a harmful algal bloom in 2011 and has not recovered [Ref.^47^, Sanford, *personal observations*]. The barnacle *Semibalanus cariosus*, a northern species, declined precipitously from 162 individuals (1941) to only one individual (2019). Two primarily southern species (the chiton *Mopalia lionata* and the mussel *Modiolus carpenteri*) were not recorded in 1941 but had one and two individuals in the 2019 survey, respectively. Lastly, 22 of the cosmopolitan species (i.e., those with broad geographic ranges; see Methods) increased in abundance, 11 decreased, and one showed no change.

**Table 1:** Species list used to analyze how mussel bed communities have changed at Dillon Beach, California.

				Temporal Comparison			Spatial Comparison (2019)			
	Species or Taxon	Class	Range	1941	2019		F&H Subsample	Plot 1	Plot 2	Plot 3
Annelida										
	*Arabella iricolor*	Polychaeta	C	8	29		20	49	1	51
	*Eulalia quadrioculata*	Polychaeta	C	0	0		0	6	0	2
	*Hemipodia simplex*	Polychaeta	C	0	1		1	1	0	0
	Lumbrineridae	Polychaeta	C	2	1		0	0	0	0
	Nereididae	Polychaeta	C	87	12		5	7	8	122
	Oenonidae	Polychaeta	C	0	4		1	0	0	1
	Orbiniidae	Polychaeta	C	0	2		2	127	1	119
	*Pherusa andersonorum*	Polychaeta	C	0	1		1	0	0	0
	*Phragmatopoma californica*	Polychaeta	S	58	33		7	59	15	98
	Polynoidae	Polychaeta	C	0	0		0	0	2	1
	Sabellidae	Polychaeta	C	1	0		0	0	0	2
	Syllidae	Polychaeta	C	67	196		78	44	81	72
**Sipuncula**										
	*Phascolosoma agassizii*	Phascolosomatidea	C	5	63		44	76	17	148
**Nemertea**										
	*Amphiporus* “*imparispinosus*” complex	Hoplonemertea	C	0	56		30	2	10	18
	*Emplectonema viride*	Hoplonemertea	C	7	8		5	1	61	16
	*Paranemertes* “*peregrina*” complex	Hoplonemertea	C	16	27		13	8	1	2
**Arthropoda**										
	Amphipoda	Malacostraca	C	142	148		21	13	17	275
	*Cirolana harfordi*	Malacostraca	C	795	111		36	8	1	2008
	*Fabia subquadrata*	Malacostraca	C	3.75	0		0	0.562	0	0.855
	*Hemigrapsus oregonensis*	Malacostraca	C	0	5		1	3	2	0
	*Pachygrapsus crassipes*	Malacostraca	C	12	44		15	8	5	1
	*Pachycheles rudis*	Malacostraca	C	0	0		0	0	0	47
	*Pagurus* sp.	Malacostraca	C	0	0		0	0	0	2
	*Pentidotea wosnesenskii*	Malacostraca	N	12	0		0	0	0	2
	*Petrolisthes cinctipes*	Malacostraca	N	695	220		125	120	15	455
	*Anoplodactylus viridintestinalis*	Pycnogonida	C	0	2		1	1	1	1
	*Pycnogonum stearnsi*	Pycnogonida	C	0	0		0	0	3	2
	Acorn barnacles (*Balanus glandula* & *Chthamalus dalli*)	Thecostraca	C	37,000	17,940		3583	10,869	15,418	14,334
	*Pollicipes polymerus*	Thecostraca	C	398	96		49	60	466	384
	*Semibalanus cariosus*	Thecostraca	N	162	1		0	0	1	0
**Cnidaria**										
	*Anthopleura elegantissima*	Anthozoa	C	17	9		7	15	16	8
**Echinodermata**										
	*Cucumaria pseudocurata*	Holothuroidea	N	79	0		0	0	0	0
**Mollusca**										
	*Adula californiensis*	Bivalvia	C	0	10		1	4	47	18
	*Leukoma staminea*	Bivalvia	C	0	1		0	0	0	0
	*Modiolus carpenteri*	Bivalvia	S	0	2		1	2	2	2
	*Mytilus californianus*	Bivalvia	C	4652	14,220		4955	5567	4204	2852
	*Amphissa versicolor*	Gastropoda	C	0	0		0	0	0	2
	*Lacuna marmorata*	Gastropoda	C	0	2		1	1	1	0
	*Littorina plena*	Gastropoda	C	4	16		0	3	13	8
	*Lottia digitalis*	Gastropoda	C	159	67		12	4	102	4
	*Lottia pelta*	Gastropoda	C	836	204		50	143	315	562
	*Lottia scabra*	Gastropoda	S	4	35		9	2	3	0
	*Lottia* sp.	Gastropoda	C	145	34		2	0	7	19
	*Nucella canaliculata*	Gastropoda	N	20	4		2	3	15	80
	*Nucella ostrina*	Gastropoda	N	84	30		8	33	18	160
	*Tegula funebralis*	Gastropoda	C	90	627		174	399	67	360
	*Cyanoplax dentiens*	Polyplacophora	C	8	77		5	1	3	0
	*Mopalia lionota*	Polyplacophora	S	0	1		1	1	0	0
	*Mopalia muscosa*	Polyplacophora	C	1	1		1	1	1	9
	*Nuttallina californica*	Polyplacophora	C	0	1		0	0	1	0

Values are the number of individuals recorded in each plot. The temporal data compare abundances in the original large 0.70 m^2^ mussel bed plot surveyed in 1941 and 2019. A spatial comparison of four 0.16 m^2^ replicate plots surveyed in 2019 was also performed, with one of the replicates (F&H Subsample) being a subsample of the plot used in the temporal comparison. Geographic range classified as S = southern, N = northern, C = cosmopolitan (see methods for details).

## Discussion

### Historical changes in a mussel bed community

What conclusions can be drawn about ecological changes in this mussel bed community? Without more intensive historical sampling and replication of the baseline data, we cannot conclude with much confidence whether a given species of interest has changed in abundance over this 78-year period. Nonetheless, aggregate patterns emerge at the level of the entire data set, and two robust conclusions can be drawn. First, there is no evidence of a decline in species diversity in mussel beds at this study site in northern California. Secondly, relative to 1941, the abundance of northern species has declined, whereas the abundance of most southern species has increased.

Contrary to our expectation, these data provide no support for the hypothesis that species diversity has declined in mussel beds at Dillon Beach. Only one species disappeared between the 1941 and 2019 surveys. Species richness was greater in 2019 than in 1941(40 species versus 31 species, respectively; Fig. 3a). Most of the additional species recorded in 2019 were uncommon species found in low abundance. We cannot completely rule out that similarly uncommon species were present but overlooked by Fisher and Hildebrand^46^. However, at a minimum, our results suggest that species richness has not decreased compared to the initial sampling ~ 8 decades ago. In contrast, Smith et al.^41^ documented a mean loss of 58.9% of species richness in mussel bed communities over ~ 15–30 years, primarily at sites in southern California (south of Point Conception).

Hypothesized drivers of the declines in diversity in southern California include a decrease in the areal extent of mussel beds^48,49^ and that mussel beds have transitioned from multi-layer beds to single layer beds^40,50^. In contrast, mussel beds at Dillon Beach, and almost all sites that we have surveyed in northern California and Oregon, generally have multiple layers^51^; Longman and Sanford, *personal observation*). Although we have historical data only from the Dillon Beach site, our surveys of mussel bed fauna at nearby Bodega Head (~ 11 km northwest of Dillon Beach) reveal a similarly high level of biodiversity (Sanford, *unpublished data*). Smith et al.^41^ hypothesized that the deterioration of mussel beds in southern California was likely related to climate change, especially declines in primary productivity and increases in ocean temperature. Mussel beds in northern California may be proportionally less impacted if the magnitude of oceanographic change has been less than in southern California. The California coast is marked by the seasonal upwelling of cold, nutrient-rich water that fuels high primary productivity. In these systems, climate change is causing upwelling intensification in poleward regions and is predicted to cause increased productivity in poleward regions and decreased productivity in equatorial regions^52^. In the California Current during the last two decades, there has been a large contraction in the spatial extent of cool sea surface temperatures associated with upwelling in southern California, while only modest seasonal contraction has occurred in northern California^53^. This has been accompanied by an increase in surface chlorophyll-a concentration in central California and a decrease in productivity in the central North Pacific Gyre off southern Baja California. However, thus far, there are no apparent differences in productivity between northern and southern California^54^. In addition, warming of waters adjacent to the shoreline has been documented, with an increase of 1.24 °C per century in surface waters and 1.67 °C per century in bottom waters at the end of Scripps Pier in San Diego, California^55^. Other anthropogenic impacts known to impact mussel beds, including pollution, harvesting, and human trampling^56–59^, might also contribute to regional differences in the health of mussel beds as these factors are likely less severe north of San Francisco, where the coastline is generally less urbanized than many areas of southern California.

**Fig. 6:**
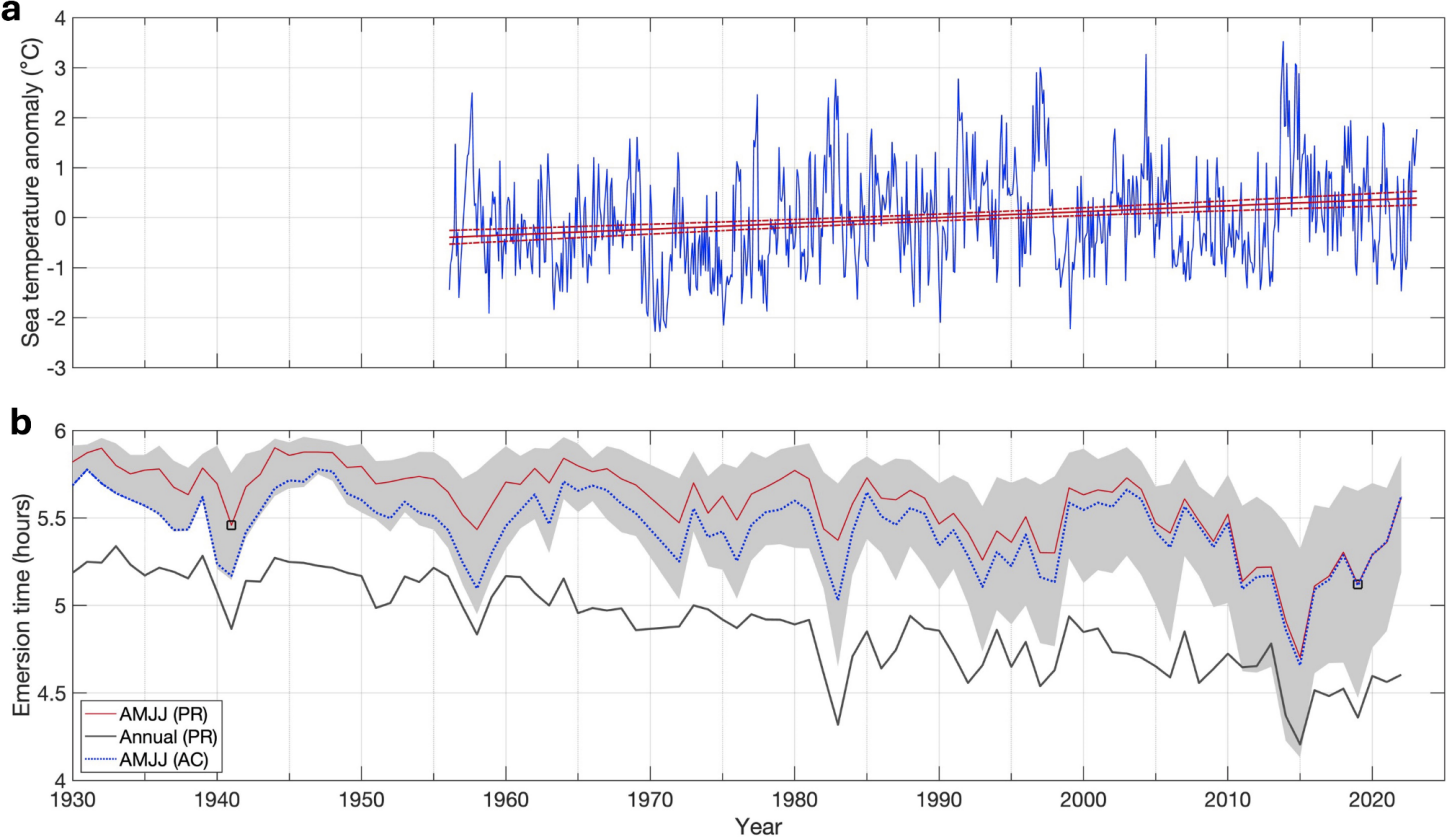
(**a**) Monthly water temperature anomalies based on Bodega Head measurements taken daily at ~ 8:00am from 1956 to 2023. The long-term trend and 95% confidence interval are shown in red. (**b**) Average emersion time (time out of water) at Point Reyes (PR) and Arena Cove (AC), California, for the April-July time frame (AMJJ) and annually at a level of 1.38 m above Mean Lower Low Water (equal to -0.008 m relative to NAVD-88 datum) from 1930–2022, for the period between − 2 h and + 4 h of solar noon. The shading represents emersion time variability for ±0.1 m of the 1.38 m level. The boxes highlight the emersion time during the 1941 and 2019 surveys.

The second major conclusion from this research is that shifts in the relative abundance of northern versus southern species between 1941 and 2019 are consistent with predicted community responses to warming temperatures. Comparable with warming at the Scripps Pier in San Diego^55^, mean monthly water temperature anomalies recorded at Bodega Head (calculated as monthly mean temperatures minus the monthly climatological temperature) increased by 0.117 ± 0.036 °C per decade between 1956 and 2023 (Fig. 6a). Warming may be weaker offshore and closer to upwelling centers like Point Arena, but warming at Dillon Beach may be stronger given the influence of warm tidal outflow from Tomales Bay and the likelihood that bay waters have warmed faster than coastal waters cooled by upwelling [^60^, Speiser and Largier, *unpublished data*]. If species are tracking this observed oceanographic warming (i.e., Fig. 6a), their geographic ranges are predicted to shift poleward, leading to an increased abundance of southern (warm-adapted) species and a decreased abundance of northern (cool-adapted) species^36,44^. Our findings indicate that all 6 of the northern species have declined in abundance since 1941, whereas 3 of the 4 southern species increased in abundance (Fig. 5). This result is consistent with prior work showing that many marine species are shifting their ranges poleward with climate change [e.g., ^36,45,61,62^]. In addition to the survey of the mussel bed habitat, Fisher and Hildebrand^46^ also included an overall species list for several other intertidal habitats at this field site. Including these habitats, we qualitatively find that many other southern species that were unrecorded or occurred in very low abundance in 1941^63^ are now present and sometimes relatively common at Dillon Beach, such as the owl limpet *Lottia gigantea*, the porcelain crab *Petrolisthes manimaculis*, the barnacle *Tetraclita rubescens*, and the nudibranch *Phidiana hiltoni* [^45^,^64,65^, Sanford and Sones, *unpublished data*].

Besides ocean warming, shifts in emersion time are an alternative hypothesis that might contribute to changes in the relative abundance of warm versus cool-adapted species. Previous research indicates that emersion times in the intertidal zone can vary substantially with an 18.6-year lunar cycle, leading to shifts in the vertical distribution of mussels^66,67^. If the two surveys had been performed during different phases of the 18.6-year oscillation then the long-term trend in species abundances could be confounded by this periodic fluctuation^66^. This is because an increase in emersion times represents an increase in exposure to aerial conditions, which might favor a shift towards warm-adapted species from upper intertidal zones or southern biogeographic regions. However, a combination of tidal predictions and available water level data from Point Reyes (~ 37 km south of the study site) and Arena Cove (~ 98 km northwest) exhibits a slight downward trend in daytime emersion time during spring (April–June) from 5.2 to 5.8 h per day in the 1930–1940s to about 4.7 to 5.6 h per day over the past decade, for the level of the lower edge of the mussel bed plot at Dillon Beach (~ 1.38 m MLLW; Fig. 6b). Estimated emersion times for Arena Cove decrease less than Point Reyes because of a smaller relative sea-level rise increase (1.08 ± 0.4 mm/year versus 2.08 ± 0.43 mm/year, respectively, from 1978 to 2023). A similar decrease in emersion times is expected at this study site, driven by a similar rate of sea-level rise. Interannual minima in emersion time are related to elevated water levels observed during the 1940–41, 1958, 1982–1983, and 2015–2016 El Niño events. Because of similar tidal forcing and anomalously high sea-levels in 1941, the estimated emersion time was only slightly lower in 2019 (5.1 h) than in 1941 (5.2–5.5 h, using Arena Cove and Point Reyes based estimates, respectively). The observed trend in emersion time is in the opposite direction than one would predict to favor an increase in southern/warm-adapted species. We acknowledge that we lack data on changes in air temperature, wave forcing and wind conditions, which could have increased levels of physical stress during low tides despite the long-term trend in decreased emersion time. Nevertheless, available data are consistent with the hypothesis that the observed changes in species composition are most strongly associated with an increase in water temperature (Fig. 6a).

## Challenges of historical studies in ecology

This study provides an opportunity to explore how useful information can be gleaned from historical data that are often characterized by several limitations. Most importantly, there was no spatial replication in the baseline sampling conducted in 1941; only a single large plot was sampled. If spatial variation among nearby mussel bed plots within a site exceeded differences observed over time at a single plot, small-scale spatial variation would likely overwhelm any temporal signal. However, our 2019 sampling suggested that spatial variation in species richness among replicate plots was relatively low in this community (Fig. 3b). Although there was some variation in the identity of species in each plot, ~ 72% of the species were common to all four spatial replicate plots. The species that differed among the four plots were generally uncommon species that occurred in low abundance. Thus, our results suggest that sampling a single large plot, although clearly not a recommended sampling design, provided a representative estimate of species diversity within mussel beds at this site.

A second major limitation was that there were only two surveys conducted 78 years apart. Thus, we do not know if samples during intervening years might have shown different trends or fluctuations due to natural variability. Of particular concern when sampling only two time points is that differences between community samples might be disproportionately influenced by short-term extreme events that occurred near the time of the surveys^44^. For example, in the northeast Pacific, periodic El Niño events typically last for 9–12 months and are characterized by warm ocean conditions, higher sea-level, and anomalous currents that can lead to an influx of primarily southern species into northern California^45^. In this study, both the initial and recent surveys were conducted within three years of a strong El Niño. The initial survey in May–June 1941 was in the midst of the strong and extended 1940–1942 El Niño event^68^, whereas the 2019 sampling was approximately 2.5 years after the very strong 2015–2016 El Niño event^45^. Thus, it is unlikely that differences in proximity to El Niño events can explain the shifts in the relative abundance of northern and southern species documented in our 1941 versus 2019 comparison. If the abundance of warm-adapted species was increased temporarily by El Niño conditions, those effects should have been more pronounced for the 1941 survey, which was conducted in the middle of the strong El Niño event.

Another possible confounding factor would be if the mussel bed structure differed strongly between 1941 and 2019. The density, size, and three-dimensional complexity of foundation species can produce strong effects on the community of organisms found within habitats created by corals, kelp, seagrasses, mussels, and other foundation species^69^. Mussel bed structure could differ between the 1941 and 2019 sample if, for example, the plot had been more recently disturbed before one of the sampling periods (i.e., returning the community to an earlier stage of succession with less habitat complexity^70^). Despite some differences in the total number of mussels in the 1941 and 2019 surveys, the size frequency distributions were relatively similar in both years and were dominated by small mussels (< 20 mm long; Fig. 2a). The total number of mussels in 2019 was three times greater than in 1941, and this was mostly due to the greater abundance of small mussels (< 30 mm long; Appendix S2: Table S2) in the latter survey, an indicator of high larval recruitment. Although the 1941 mussel bed included a greater proportion of large mussels (60–110 mm long), some mid- and large-sized mussels were present in both years, indicating a mature mussel bed that had not been recently disturbed. In addition, our spatial comparison in 2019 indicates that, despite some variation in the size and structure of the mussel beds surveyed, the community composition was relatively similar across the four spatial replicates. This gives us confidence that the organisms living within the mussel matrix are not strongly influenced by modest changes in the structure of the mussel bed. With regards to epibionts living on mussel shells, the valves of large mussels often attract heavy barnacle recruitment, so the smaller number of large mussels in 2019 may have contributed to the apparent decline in barnacle abundance in the historical plot.

Several qualitative factors further increase our confidence in this historical comparison. First, we were able to relocate and resample the exact same large plot as in 1941, thus minimizing potential spatial differences in local factors known to influence diversity in mussel beds such as vertical tidal height, wave exposure, or substratum orientation^71–74^. Second, these unpublished data were quantitative and generally had high taxonomic resolution. All individuals within the plot were counted, and the sampling was supervised by Professor S.F. Light, the preeminent invertebrate zoologist in California at the time. Thus, we are confident that species were identified correctly in the baseline data.

## Conclusions

While historical data often have limitations, these unpublished and non-traditional resources can yield important ecological insights^9,10,12^. As many ecosystems are changing rapidly, using historical documents is often one of the only ways to evaluate the magnitude of change that has occurred over the last several decades to century. Our study illustrates several ways that researchers can address the limitations of historical data sets to make more robust inferences [see also ^12^,^75^]. Although non-traditional data sets have been underutilized in marine research^10^, historical ecology has the potential to help address how marine communities have changed relative to baseline communities of the past^12,22^.

### Methods

#### Historical report and survey methods

The foundation for this study was an unpublished 1941 graduate student report discovered in the Cadet Hand Library collection at Bodega Marine Laboratory^46^. The students’ survey of the mussel bed community at Dillon Beach, California, was particularly thorough and entailed identifying and counting 45,953 individual organisms, as well as measuring and analyzing the size structure of the mussel bed. The project was supervised by Dr. S.F. Light, a well-known zoologist who authored the first edition of the primary taxonomic key that is still used to identify marine invertebrates in this region^76^.

Our mussel bed survey in 2019 consisted of both temporal and spatial comparisons. First, the exact mussel bed plot at Dillon Beach from 1941 was relocated (coordinates: 38°15’27.2” N, -122°58’16.7” W, World Geodetic System 1984) and resurveyed. We sampled this large plot (quadrat: 2.5 ft wide x 3 ft tall = 76 cm wide x 92 cm tall = 0.70 m^2^) using the same methods as in the 1941 study; all mussels and other organisms within the quadrat were removed with hand tools, and species were then identified and counted in the laboratory. Thus, species abundances (i.e., the number of individuals per 0.70 m^2^) are directly comparable between 1941 and 2019.

Additionally, in 2019, we sampled four replicate mussel bed plots (quadrat: 40 × 40 cm = 0.16 m^2^). One of the replicates, “F&H Subsample”, was a subsample of the larger plot used in the temporal comparison. Our spatial comparison tested whether local plot-to-plot variation was large enough to overwhelm detection of any temporal patterns of change. All sample locations were from roughly the same tidal elevation (upper mid-intertidal mussel beds) within the same boulder field (replicate plots were separated by ~ 40 m). The mussel beds were surveyed in May to August of 2019, a similar time of year to the 1941 study conducted in May to June, minimizing any effects of seasonal changes in species presence and abundance. Although we selected replicate mussel beds of similar tidal height and wave exposure, some aspects of the mussel beds surveyed in 2019 varied among replicate plots including mussel density, size structure, and bed depth. In addition to measuring mussel sizes (see below), the depth of each mussel bed was measured in multiple locations (*n* = 10–16) within the plot before any organisms were removed.

After samples were brought back to Bodega Marine Laboratory, organisms were sorted, counted, and identified to the species level, or the lowest taxonomic level possible, using regional taxonomic keys^76^. For species with uncertain identification, photographs were taken using a dissecting microscope (Leica MC170 HD camera attached to a Leica M125 microscope) and preserved for consultation with taxonomic experts (see Acknowledgements). Polychaete worms were preserved in either 95% ethanol or 4% formalin then transferred to 70% ethanol and deposited at the Natural History Museum of Los Angeles County. When the small brooding clam *Lasaea subviridis* was common, we subsampled the sediment to estimate totals. To quantify the number of pea crabs (*Fabia subquadrata*) present within 100 mussels, medium to large mussels (*M. californianus*) were dissected from each plot (*n* = 62–234 per plot, sample size varied due to variation in the number of medium and large mussels across plots). To compare changes in mussel size distribution across space and time, all live mussels from the plots were measured along their anterior-posterior axis using calipers. Mussels less than 10 mm in length were not individually measured, but were instead grouped into 0–5 mm, and 5–10 mm bins. Barnacle estimates were determined based on both those attached to the underlying rocks and mussels, using a subsampling approach. Additional sampling details are in Appendix S2: Sect. 1.

### Analyses

All statistical analyses were performed in R (version 3.5.1)^77^. A Wilcoxon test was performed to assess if the mean of the size frequency distribution of mussels differed between time periods. The 1941 survey only included data in size bins and in 2019 mussels less than 10 mm in length were not individually measured; thus, mussels in these size classes were randomly assigned a length using a uniform distribution within their respective size bin. A Kruskal-Wallis test was used to assess differences in the mean mussel size across the spatial replicates. Chi-square tests were used to compare the mussel bed size bin data between time periods and among spatial replicates. Lastly, a linear model was used to compare the depth of the mussel bed among the four spatial replicates. Assumptions were analyzed visually, and normality was evaluated with a Shapiro-Wilk test.

As a resource for potential surveys of mussel beds at this site in the future, we include our complete species list with as much taxonomic detail as available (Appendix S1). Our analyses only included species that were attached and living within the mussel bed. This excluded bryozoans and hydroids that were collected, but were likely dislodged animals that had washed into the bed from lower tidal zones.

We updated the species names for the 1941 historical species list so that data would be comparable to modern taxonomic keys. In a few cases, a species was described after 1941, thus we grouped species as needed to facilitate comparison (Appendix S2: Table S1). For example, *Lottia paradigitalis* was not described until 1960^78^, so we grouped *L. paradigitalis* with *L. digitalis* for our analyses. We compared the lowest taxonomic group possible between the two time periods. This required further grouping some species in the 2019 data to be comparable to the survey in 1941 (Appendix S2: Table S1). We omitted many small and inconspicuous species from our species list (e.g., oligochaetes, microgastropod species that are less than 2–3 mm long, etc.), as the 1941 study did not include these very small species (Appendix S2: Table S1). We also dropped a few species that were noticeably absent from the historical survey. For example, Fisher and Hildebrand^46^ apparently excluded flatworms and sponges in their mussel bed survey, although these taxa were mentioned elsewhere in their surveys of other habitats at this site, and were known to be present on rocky shores during this time^63^.

Species richness, diversity (Shannon-Wiener Index) and evenness (Pielou’s Index) were compared between the two time periods and among the four spatial replicates. To determine if the species composition of mussel beds has shifted based on biogeographic affinities, species were classified by their geographic range boundaries. “Northern” species were species considered to be cool-adapted that have a southern range boundary near or north of Point Conception, California^44^. “Southern” species were species considered to be warm-adapted that have a northern range boundary near or south of Coos Bay, Oregon. “Cosmopolitan” species were species with boundaries extending beyond these geographic boundaries in both directions^44^. We compared log (abundance + 1) between the two time periods to analyze these shifts in community composition.

### Oceanographic analyses – water temperature and sea level

Coastal water temperature from 1956 to 2023 was collected at Bodega Head, ~ 11 km north of Dillon Beach. Measurements were initially collected daily around 8:00am, and since 1989 measurements were recorded with a high-precision electronic thermistor on the seawater intake for Bodega Marine Laboratory, which draws water from Horseshoe Cove on Bodega Head. Automated measurements were quality controlled following^79^ and the measurement closest to 8:00am each day was extracted to create a consistent time series for the entire time period. A monthly climatology was calculated from these data (Appendix S2: Fig. S3) and a monthly anomaly time series was obtained from monthly mean temperatures minus monthly climatological temperature (Fig. 6a). The long-term trend in temperature is obtained by fitting a linear trend to the monthly anomaly values.

Water levels and emersion times were evaluated from 1930 to 2022 using tidal data series at Point Reyes and Arena Cove, located ~ 37 km south and ~ 98 km northwest of Dillon Beach, respectively [National Oceanographic and Atmospheric Administration (NOAA) stations 9415020 and 9416841]. Available hourly records were linearly interpolated to 6-minute frequency, and the average daily emersion time for different vertical elevations was calculated over the time period of peak solar forcing, which we defined as between − 2 h and + 4 h of solar noon (solar noon is the time that the sun reaches its daily zenith and varies by longitude and time of year). For 1930–1974 (Point Reyes) and 1930–1977 (Arena Cove), emersion times were estimated using a synthetic data set which combined local tidal predictions with the non-tidal residual (NTR; defined as measured minus predicted tides) from the nearest available measurement at the San Francisco tide gauge (NOAA gauge 9414290). Relative sea-level rise rates were extrapolated linearly to 1930 using a robust least-squares fit to annual sea-level over the period of record (1975–2023 for Point Reyes and 1978–2023 for Arena Cove). Tidal predictions were obtained using the Utide harmonic analysis package^80^. The use of the San Francisco NTR enabled us to estimate the influence of interannual sea-level variability and seasonal upwelling [see e.g., ^81^] on historical emersion time; nonetheless, occasional small non-oceanic influences on water levels are introduced through river-flow effects^82,83^.

## Electronic supplementary material

Below is the link to the electronic supplementary material.

Supplementary Material 1

Supplementary Material 2

## Data Availability

The ecological dataset for this study is provided in Appendix S1. Additional datasets are available upon request from the corresponding author.

## References

[1] Butchart, S. H. et al. Global biodiversity: indicators of recent declines. *Science* **328**, 1164–1168 (2010). 20430971 10.1126/science.1187512

[2] Díaz, S. et al. Pervasive human-driven decline of life on Earth points to the need for transformative change. *Science* **366**, eaax3100. 10.1126/science.aax3100 (2019). 31831642 10.1126/science.aax3100

[3] Jaureguiberry, P. et al. The direct drivers of recent global anthropogenic biodiversity loss. *Sci. Adv.* **8**, eabm9982. 10.1126/sciadv.abm9982 (2022). 36351024 10.1126/sciadv.abm9982 PMC9645725

[4] Cardinale, B. J. et al. Biodiversity loss and its impact on humanity. *Nature* **486**, 59–67 (2012). 22678280 10.1038/nature11148

[5] McCauley, D. J. et al. Marine defaunation: animal loss in the global ocean. *Science* **347**, 1255641. 10.1126/science.1255641 (2015). 25593191 10.1126/science.1255641

[6] Loreau, M., Naeem, S. & Inchausti, P. (eds) *Biodiversity and Ecosystem Functioning: Synthesis and Perspectives* (Oxford University Press, 2002).

[7] Rick, T. C. & Lockwood, R. Integrating paleobiology, archeology, and history to inform biological conservation. *Conserv. Biol.* **27**, 45–54 (2013). 22979917 10.1111/j.1523-1739.2012.01920.x

[8] Lister, A. M. & Climate Change Research Group. Natural history collections as sources of long-term datasets. *Trends Ecol. Evol.* **26** (4), 153–154 (2011). 21255862 10.1016/j.tree.2010.12.009

[9] Thurstan, R. H. et al. Filling historical data gaps to foster solutions in marine conservation. *Ocean. Coast Manag*. **115**, 31–40 (2015).

[10] Beller, E. E., McClenachan, L., Zavaleta, E. S. & Larsen, L. G. Past forward: recommendations from historical ecology for ecosystem management. *Glob Ecol. Conserv.* **21**, e00836. 10.1016/j.gecco.2019.e00836 (2020).

[11] McClenachan, L., Ferretti, F. & Baum, J. K. From archives to conservation: why historical data are needed to set baselines for marine animals and ecosystems. *Conserv. Lett.* **5**, 349–359 (2012).

[12] Sagarin, R. & Pauchard, A. *Observation and Ecology: Broadening the Scope of Science to Understand a Complex World* (Island, 2012).

[13] Beller, E. et al. Toward principles of historical ecology. *Am. J. Bot.* **104**, 645–648 (2017). 28515077 10.3732/ajb.1700070

[14] Swetnam, T. W., Allen, C. D. & Betancourt, J. L. Applied historical ecology: using the past to manage for the future. *Ecol. Appl.* **9**, 1189–1206 (1999).

[15] Boggess, W. R. & Bailey, L. W. Brownfield Woods, Illinois: Woody vegetation and changes since 1925. *Am. Midl. Nat.* **71**, 392–401 (1964).

[16] Parmesan, C. et al. Poleward shifts in geographical ranges of butterfly species associated with regional warming. *Nature* **399**, 579–583 (1999).

[17] Miller-Rushing, A. J., Primack, R. B., Primack, D. & Mukunda, S. Photographs and herbarium specimens as tools to document phenological changes in response to global warming. *Am. J. Bot.* **93**, 1667–1674 (2006). 21642112 10.3732/ajb.93.11.1667

[18] Willis, C. G., Ruhfel, B., Primack, R. B., Miller-Rushing, A. J. & Davis, C. C. Phylogenetic patterns of species loss in Thoreau’s woods are driven by climate change. *Proc. Natl. Acad. Sci. USA*, 105, 17029–17033 https://doi.org/10.1073/pnas.0806446105 (2008). 10.1073/pnas.0806446105 PMC2573948 18955707

[19] Gallagher, R. V., Hughes, L. & Leishman, M. R. Phenological trends among Australian alpine species: using herbarium records to identify climate-change indicators. *Aust J. Bot.* **57**, 1–9 (2009).

[20] Tingley, M. W., Monahan, W. B., Beissinger, S. R. & Moritz, C. Birds track their Grinnellian niche through a century of climate change. *Proc. Natl. Acad. Sci. USA*, 106, 19637–19643 https://doi.org/10.1073/pnas.0901562106(2009). 10.1073/pnas.0901562106 PMC2780944 19805037

[21] Cohen, A. N. & Carlton, J. T. Accelerating invasion rate in a highly invaded estuary. *Science* **279**, 555–558 (1998). 9438847 10.1126/science.279.5350.555

[22] Jackson, J. B. et al. Historical overfishing and the recent collapse of coastal ecosystems. *Science* **293**, 629–637 (2001). 11474098 10.1126/science.1059199

[23] Sagarin, R. D., Gilly, W. F., Baxter, C. H., Burnett, N. & Christensen, J. Remembering the Gulf: changes to the marine communities of the Sea of Cortez since the Steinbeck and Ricketts expedition of 1940. *Front. Ecol. Environ.* **6**, 374–381 (2008).

[24] Sorte, C. J. & Stachowicz, J. J. Patterns and processes of compositional change in a California epibenthic community. *Mar. Ecol. Prog Ser.* **435**, 63–74 (2011).

[25] Bertness, M. D. & Callaway, R. Positive interactions in communities. *Trends Ecol. Evol.* **9**, 191–193 (1994). 21236818 10.1016/0169-5347(94)90088-4

[26] Angelini, C., Altieri, A. H., Silliman, B. R. & Bertness, M. D. Interactions among foundation species and their consequences for community organization, biodiversity, and conservation. *BioSci* **61**, 782–789 (2011).

[27] Waycott, M. et al. Accelerating loss of seagrasses across the globe threatens coastal ecosystems. *Proc. Natl. Acad. Sci. USA*. **106**, 12377–12381 (2009). 19587236 10.1073/pnas.0905620106 PMC2707273

[28] Smale, D. A. & Wernberg, T. Extreme climatic event drives range contraction of a habitat-forming species. *Proc. R. Soc. B: Biol. Sci.*, 280, 20122829 10.1098/rspb.2012.2829 (2013). 10.1098/rspb.2012.2829 PMC3574333 23325774

[29] Sorte, C. J. et al. Long-term declines in an intertidal foundation species parallel shifts in community composition. *Glob Chang. Biol.* **23**, 341–352 (2017). 27411169 10.1111/gcb.13425

[30] Eddy, T. D. et al. Global decline in capacity of coral reefs to provide ecosystem services. *One Earth*. **4**, 1278–1285 (2021).

[31] O’Donnell, M. J. Reduction of wave forces within bare patches in mussel beds. *Mar. Ecol. Prog Ser.* **362**, 157–167 (2008).

[32] Jurgens, L. J., Ashlock, L. W. & Gaylord, B. Facilitation alters climate change risk on rocky shores. *Ecology* **103**, e03596. 10.1002/ecy.3596 (2022). 34813668 10.1002/ecy.3596

[33] Witman, J. D. Refuges, biological disturbance, and rocky subtidal community structure in New England. *Ecol. Monogr.* **55**, 421–445 (1985).

[34] Gutiérrez, J. L., Jones, C. G., Strayer, D. L. & Iribarne, O. O. Mollusks as ecosystem engineers: the role of shell production in aquatic habitats. *Oikos* **101**, 79–90 (2003).

[35] Lafferty, K. D. & Suchanek, T. H. Revisiting Paine’s 1966 sea star removal experiment, the most-cited empirical article in the American naturalist. *Am. Nat.* **188**, 365–378 (2016). 27622872 10.1086/688045

[36] Sunday, J. M. et al. Ocean acidification can mediate biodiversity shifts by changing biogenic habitat. *Nat. Clim. Change*. **7**, 81–85 (2017).

[37] Sampaio, L., Moreira, J., Rubal, M., Guerrero-Meseguer, L. & Veiga, P. A review of coastal anthropogenic impacts on Mytilid mussel beds: effects on mussels and their associated assemblages. *Diversity* **14**, 409. 10.3390/d14050409 (2022).

[38] Kanter, R. G. Biogeographic patterns in mussel community distribution from the Southern California Bight in The California Islands: Proceedings of a Multidisciplinary Symposium (ed. Power, D.M.) 341–355. Santa Barbara Museum of Natural History, Santa Barbara, CA (1980).

[39] Suchanek, T. H. Extreme biodiversity in the marine environment: mussel and communities of *Mytilus californianus*. *Northwest. Environ. J.* **8**, 150–152 (1992).

[40] Smith, J. R., Fong, P. & Ambrose, R. F. Long-term change in mussel (*Mytilus californianus* Conrad) populations along the wave-exposed coast of southern California. *Mar. Biol.* **149**, 537–545 (2006).

[41] Smith, J. R., Fong, P. & Ambrose, R. F. Dramatic declines in mussel bed community diversity: response to climate change? *Ecology* **87**, 1153–1161 (2006). 16761594 10.1890/0012-9658(2006)87[1153:ddimbc]2.0.co;2

[42] Fales, R. J. & Smith, J. R. Long-term change in a high-intertidal rockweed (*Pelvetiopsis Californica*) and community-level consequences. *Mar. Biol.* **169**, 34. 10.1007/s00227-022-04022-1 (2022).

[43] Barry, J. P., Baxter, C. H., Sagarin, R. D. & Gilman, S. E. Climate-related, long-term faunal changes in a California rocky intertidal community. *Science* **267**, 672–675 (1995). 17745845 10.1126/science.267.5198.672

[44] Sagarin, R. D., Barry, J. P., Gilman, S. E. & Baxter, C. H. Climate-related change in an intertidal community over short and long time scales. *Ecol. Monogr.* **69**, 465–490 (1999).

[45] Sanford, E., Sones, J. L., García-Reyes, M., Goddard, J. H. R. & Largier, J. L. Widespread shifts in the coastal biota of northern California during the 2014–2016 marine heatwaves. *Sci. Rep.* **9**, 4216. 10.1038/s41598-019-40784-3 (2019). 30862867 10.1038/s41598-019-40784-3 PMC6414504

[46] Fisher, H. I. & Hildebrand, M. The fauna of a selected rocky shore: a qualitative and quantitative study of the fauna of the rocks of second Sled Road Point, Dillon Beach, Marin County, California. University of California Berkeley, Zoology S119, Student Report. Cadet Hand Library, Bodega Marine Laboratory (1941).

[47] Jurgens, L. J. et al. Patterns of mass mortality among rocky shore invertebrates across 100 km of northeastern Pacific coastline. *PLOS ONE*. **10**, e0126280. 10.1371/journal.pone.0126280 (2015). 26039349 10.1371/journal.pone.0126280 PMC4454560

[48] Engle, J. M. & Davis, G. E. *Ecological Condition and Public Use of the Cabrillo National Monument Intertidal Zone 1990–1995* (US Department of the Interior, US Geological Survey, 2000).

[49] Engle, J. M. & Davis, G. E. *Baseline Surveys of Rocky Intertidal Ecological Resources at Point Loma, San Diego* (US Department of the Interior, US Geological Survey, 2000).

[50] Robles, C. D., Engle, J., Garza, C. & Becker, B. J. *Preliminary Evidence of the Collapse of Mussel beds (Mytilus californianus) in the Southern California Bight* (Western Society of Naturalists published abstract, 2015).

[51] Sanford, E. & Worth, D. J. Genetic differences among populations of a marine snail drive geographic variation in predation. *Ecology* **90**, 3108–3118 (2009). 19967866 10.1890/08-2055.1

[52] Bograd, S. J. et al. Climate change impacts on eastern boundary upwelling systems. *Ann. Rev. Mar. Sci.* **15**, 303–328 (2023). 35850490 10.1146/annurev-marine-032122-021945

[53] García-Reyes, M. et al. Most eastern boundary upwelling regions represent thermal refugia in the age of climate change. *Front. Mar. Sci.* **10**, 1158472. 10.3389/fmars.2023.1158472 (2023).

[54] Kahru, M., Kudela, R. M., Manzano-Sarabia, M. & Mitchell, B. G. Trends in the surface chlorophyll of the California Current: merging data from multiple ocean color satellites. *Deep Sea Res. Part. II: Trop. Stud. Oceanogr.* **77**, 89–98 (2012).

[55] Rasmussen, L. L. et al. A century of Southern California coastal ocean temperature measurements. *J. Geophys. Res. Oceans*, 125, e; (2019). JC015673 10.1029/2019JC015673 (2020).

[56] Smith, J. R. & Murray, S. N. *The Effects of Bait Collection and Trampling on Mytilus californianus Communities in the Southern California Intertidal zone*. Doctoral dissertation, MS Thesis, Department of Biological Sciences, California State University, Fullerton (2002).

[57] Smith, J. R. & Murray, S. N. The effects of experimental bait collection and trampling on a *Mytilus californianus* mussel bed in southern California. *Mar. Biol.* **147**, 699–706 (2005).

[58] Van De Werfhorst, L. C. & Pearse, J. S. Trampling in the rocky intertidal of central California: a follow-up study. *Bull. Mar. Sci.* **81**, 245–254 (2007).

[59] Mendez, M. M., Livore, J. P. & Bigatti, G. Effects of trampling on intertidal mussel beds: importance of disturbance intensity. *Mar. Ecol. Prog Ser.* **606**, 231–235 (2018).

[60] Roughan, M. A. et al. Subsurface recirculation and larval retention in the lee of a small headland: a variation on the upwelling shadow theme. *J. Geophys. Res.* **110**, C100027. 10.1029/2005JC002898 (2005).

[61] Poloczanska, E. S. et al. Global imprint of climate change on marine life. *Nat. Clim. Change*. **3**, 919–925 (2013).

[62] Poloczanska, E. S. et al. Responses of marine organisms to climate change across oceans. *Front. Mar. Sci.* **62**10.3389/fmars.2016.00062 (2016).

[63] Light, S. F. *Laboratory and Field Text in Invertebrate Zoology* (Associated Students Store, University of California, 1941).

[64] Nielsen, E. S. et al. Pushed waves, trailing edges, and extreme events: eco-evolutionary dynamics of a geographic range shift in the owl limpet, *Lottia gigantea*. *Glob Chang. Biol.* **30**, e17414. 10.1111/gcb.17414 (2024). 39044553 10.1111/gcb.17414

[65] Goddard, J. H. et al. Heterobranch sea slug range shifts in the northeast Pacific Ocean associated with the 2015-16 El Niño. *Proc. Calif. Acad. Sci.* **65**, 107–131 (2018).

[66] Denny, M. W. & Paine, R. T. Celestial mechanics, sea-level changes, and intertidal ecology. *Biol. Bull.* **194**, 108–115 (1998). 28570845 10.2307/1543040

[67] Helmuth, B. et al. Climate change and latitudinal patterns of intertidal thermal stress. *Science* **298**, 1015–1017 (2002). 12411702 10.1126/science.1076814

[68] Brönnimann, S. et al. Extreme climate of the global troposphere and stratosphere in 1940–42 related to El Niño. *Nature* **431**, 971–974 (2004). 15496919 10.1038/nature02982

[69] Thomsen, M. S. et al. Heterogeneity within and among co-occurring foundation species increases biodiversity. *Nat. Commun.* **13**, 581. 10.1038/s41467-022-28194-y (2022). 35102155 10.1038/s41467-022-28194-y PMC8803935

[70] Paine, R. T. & Levin, S. A. Intertidal landscapes: disturbance and the dynamics of pattern. *Ecol. Monogr.* **51**, 145–178 (1981).

[71] Seed, R. Patterns of biodiversity in the macro-invertebrate fauna associated with mussel patches on rocky shores. *J. Mar. Biol. Assoc. U K*. **76**, 203–210 (1996).

[72] McQuaid, C. D., Lindsay, J. R. & Lindsay, T. L. Interactive effects of wave exposure and tidal height on population structure of the mussel *Perna perna*. *Mar. Biol.* **137**, 925–932 (2000).

[73] Hammond, W. & Griffiths, C. L. Influence of wave exposure on South African mussel beds and their associated infaunal communities. *Mar. Biol.* **144**, 547–552 (2004).

[74] Harley, C. D. Tidal dynamics, topographic orientation, and temperature-mediated mass mortalities on rocky shores. *Mar. Ecol. Prog Ser.* **371**, 37–46 (2008).

[75] Vellend, M., Brown, C. D., Kharouba, H. M., McCune, J. L. & Myers-Smith, I. H. Historical ecology: using unconventional data sources to test for effects of global environmental change. *Am. J. Bot.* **100**, 1294–1305 (2013). 23804553 10.3732/ajb.1200503

[76] Carlton, J. (ed) The Light & Smith Manual: Intertidal Invertebrates of the California and Oregon Coasts. Fourth Edition, University of California Press, Berkeley and Los Angeles (2007).

[77] R Core Team. R: A language and environment for statistical computing. R Foundation for Statistical Computing, Vienna, Austria. https://www.r-project.org/ (2021).

[78] Fritchman, H. K. *Acmaea paradigitalis* sp. nov. *Gastropoda) Veliger*. **2**, 53–57 (1960). Acmaeidae.

[79] Bushnell, M. *Manual for Real-Time Quality Control of In-situ Temperature and Salinity Data: Version 2.1* (NOAA, 2020).

[80] Codiga, D. L. *Unified Tidal Analysis and Prediction Using the UTide Matlab Functions (Tech. Rep. No. 01)* (Graduate School of Oceanography, University of Rhode Island, 2011).

[81] Chelton, D. B. & Davis, R. E. Monthly mean sea-level variability along the west coast of North America. *J. Phys. Oceanogr.* **12**, 757–784 (1982).

[82] Moftakahri, H. R., Jay, D. A., Talke, S. A., Kulkulka, T. & Bromirski, P. D. A novel approach to flow estimation in tidal rivers. *Water Resour. Res.* **49**, 1–16 (2013).

[83] Baranes, H., Dykstra, S. L., Jay, D. A. & Talke, S. A. Sea level rise and the drivers of daily water levels in the Sacramento-San Joaquin Delta. *Sci. Rep.* **13**, 22454. 10.1038/s41598-023-49204-z (2023). 38105273 10.1038/s41598-023-49204-z PMC10725870

