# Supplementary material for: Evaluating historical changes in a mussel bed community in northern California
Source: Sci Rep. 2025 Jan 14;15:1930. doi: 10.1038/s41598-025-86105-9 (PMC11733294; doi:10.1038/s41598-025-86105-9)

Appendix S2

Title: Evaluating historical changes in a mussel bed community in northern California

Authors: Emily K. Longman, Sarah Merolla, Stefan A. Talke, Nicholas Trautman, John L. Largier, Leslie Harris & Eric Sanford

Section S1: Additional Sampling Details

Barnacle counts per plot included estimates of individuals on mussel shells plus those on the underlying rocks. In the laboratory, we counted the number of each barnacle species on a subsample of mussels (7.5–22.7% of the total) from each plot across a range of mussel sizes, excluding mussels less than 10mm (since mussels of this size rarely have barnacles). This corresponded to counting barnacles on between 313 to 1,229 mussels within each plot. We then calculated a total number of barnacles living on the mussels per plot by extrapolating from the average number of barnacles present on mussels in each 5mm size bin relative to the number of mussels of that size present in that plot. Large mussels were rare, so in a few cases, mussels of a given size bin for a specific plot were not analyzed for barnacles. In these situations, we assigned these mussels an average barnacle count from size classes just larger or smaller than that size class from the same plot. In the field, we also estimated the total number of *Balanus glandula* and *Chthamalus dalli* that remained on the rock after removing all organisms. The number of barnacles on the rock was much smaller than on the mussel shells, so we obtained a visual estimate for each plot by summing the approximate number of barnacles found in each section of the quadrat. For the first plot sampled (Plot 1) we neglected to estimate the total number of barnacles left on the rock. Thus, we assigned it the average number present from the other plots.

Supplementary Table S1. Many species were grouped or omitted from the final data sets used in the spatial and temporal comparisons. Our raw data (Appendix S1) included as much information as possible about each species/taxonomic group. For our analyses, we took a conservative approach to which organisms the students would have reasonably found and been able to identify in 1941. Thus, we grouped certain species together to have similar taxonomic resolution, and dropped those species that were likely not living within the mussel bed, as well as several small and inconspicuous species that did not appear to be considered in the 1941 survey.

**Species Grouped/Lumped**	**Species Dropped**
Nereididae - grouped *Nereis vexillosa*, *Nereis zonata*, *Nereis latescans*, *Neanthes brandti*, with other Nereididae.<br><br>Lumbrineridae - grouped *Lumbrinereis zonata* with other Lumbrineridae.<br><br>Polynoidae - grouped *Halosydna brevisetosa* with Polynoidae.<br><br>Syllidae – grouped *Syllis adamantea*, *Syllis elongata* Complex, *Syllis pigmentata/provisional* sp. nov?, with other Syllidae.<br><br>Sabellidae - grouped *Sabella media* with Sabellidae.<br><br>Grouped *Lottia limatula* with *Lottia* sp.<br><br>Grouped *Lottia paradigitalis* with *Lottia digitalis.*<br><br>Grouped *Mopalia* sp. 1 with *Mopalia mucosa.*<br><br>Grouped amphipods - *Apohyale anceps*, *Ptilohyale littoralis*, *Jassa* sp., *Elasmopus rapax*, *Paramoera* sp.	Dropped bryozoans and hydroids because these animals likely washed into the plots and were not attached/living there.<br><br>Dropped flatworms and sponges. Both taxa were mentioned elsewhere in the 1941 report, but not included on the mussel bed species list. These species are very conspicuous and abundant so likely they were not included in the mussel bed survey.<br><br>Species/taxa dropped because organisms are small/inconspicuous: Oligochaeta, *Sabellaria* sp., *Neomolgus littoralis*, Insect larva, *Munna chromatocephala*, *Pancolus californiensis*, *Lasaea subviridis*, *Barleeia haliotiphila*, *Odostomia tenuisculpta*, *Runcina macfarlandi*, Nematodes/ roundworms, *Paramorea*, Pholoinae genus A species B Harris, *Notomastus* sp., *Polycirrus* sp., "*Tetrastemma*" *bilineatum*, *Hiatella arctica*.<br><br>Dropped organisms that were clearly not the focus of the 1941 study: Staphylinidae (beetles).<br><br>Species described after 1941 and thus unknown at time of original survey: *Maculaura cerebrosa* (described in 2015).

Supplementary Table S2. Size frequency distribution of mussels (*Mytilus californianus*) in the 2019 surveys compared to 1941. Values are the number of mussels in each size class, grouped into 5 mm bins. In the temporal comparison, the plot was 0.70 m^2, whereas in the spatial comparison, the plot was 0.16 m^2.

	**Temporal Comparison**		**Spatial Comparison**			
**Mussel Length Bin (mm)**	**1941 Survey**	**2019 Survey**	**F&H Subsample**	**Plot 1**	**Plot 2**	**Plot 3**
**(0,5]**	713	1367	483	410	485	1050
**(5,10]**	709	2960	1058	964	890	366
**(10,15]**	386	2953	1125	1155	745	232
**(15,20]**	304	2194	846	941	556	217
**(20,25]**	313	1598	570	626	380	165
**(25,30]**	394	1113	326	437	278	130
**(30,35]**	391	759	195	300	193	109
**(35,40]**	343	477	123	191	172	114
**(40,45]**	230	328	98	123	116	100
**(45,50]**	240	197	66	74	110	72
**(50,55]**	162	116	35	83	90	62
**(55,60]**	134	69	16	74	55	50
**(60,65]**	87	23	5	61	57	38
**(65,70]**	97	22	3	51	32	31
**(70,75]**	49	9	1	35	16	33
**(75,80]**	34	6	1	16	7	26
**(80,85]**	19	6	1	8	1	10
**(85,90]**	23	0	0	4	1	16
**(90,95]**	9	1	0	4	0	13
**(95,100]**	8	1	0	2	0	3
**(100,105]**	3	0	0	1	0	5
**(105,110]**	4	0	0	0	0	2
**(110,115]**	0	0	0	0	0	1
**(115,120]**	0	0	0	1	0	1

Supplementary Table S3. Species density values (abundance per 1.0 m^2) used to analyze changes in mussel bed communities at Dillon Beach, California. Values are scaled based on the size of the plot: 0.70 m^2 for the temporal comparison and 0.16 m^2 for the spatial comparison. One of the replicates in the spatial comparison (F&H Subsample) was a subsample of the original plot used in the temporal comparison. Geographic range classified as S = southern, N = northern, C = cosmopolitan (see Methods for details).

			Temporal Comparison		Spatial Comparison (2019)			
**Species or Taxon**	**Class**	**Range**	**1941**	**2019**	**F&H Subsample**	**Plot 1**	**Plot 2**	**Plot 3**
**Annelida**								
Arabella iricolor	Polychaeta	C	11.43	41.43	125.00	306.25	6.25	318.75
Eulalia quadrioculata	Polychaeta	C	0.00	0.00	0.00	37.50	0.00	12.50
Hemipodia simplex	Polychaeta	C	0.00	1.43	6.25	6.25	0.00	0.00
Lumbrineridae	Polychaeta	C	2.86	1.43	0.00	0.00	0.00	0.00
Nereididae	Polychaeta	C	124.29	17.14	31.25	43.75	50.00	762.50
Oenonidae	Polychaeta	C	0.00	5.71	6.25	0.00	0.00	6.25
Orbiniidae	Polychaeta	C	0.00	2.86	12.50	793.75	6.25	743.75
Pherusa andersonorum	Polychaeta	C	0.00	1.43	6.25	0.00	0.00	0.00
Phragmatopoma californica	Polychaeta	S	82.86	47.14	43.75	368.75	93.75	612.50
Polynoidae	Polychaeta	C	0.00	0.00	0.00	0.00	12.50	6.25
Sabellidae	Polychaeta	C	1.43	0.00	0.00	0.00	0.00	12.50
Syllidae	Polychaeta	C	95.71	280.00	487.50	275.00	506.25	450.00
**Sipuncula**								
Phascolosoma agassizii	Phascolosomatidea	C	7.14	90.00	275.00	475.00	106.25	925.00
**Nemertea**								
Amphiporus "*imparispinosus*" complex	Hoplonemertea	C	0.00	80.00	187.50	12.50	62.50	112.50
Emplectonema viride	Hoplonemertea	C	10.00	11.43	31.25	6.25	381.25	100.00
Paranemertes "*peregrina*" complex	Hoplonemertea	C	22.86	38.57	81.25	50.00	6.25	12.50
**Arthropoda**								
Amphipoda	Malacostraca	C	202.86	211.43	131.25	81.25	106.25	1718.75
Cirolana harfordi	Malacostraca	C	1135.71	158.57	225.00	50.00	6.25	12550.00
Fabia subquadrata	Malacostraca	C	5.36	0.00	0.00	3.51	0.00	5.34

Hemigrapsus oregonensis	Malacostraca	C	0.00	7.14	6.25	18.75	12.50	0.00
Pachygrapsus crassipes	Malacostraca	C	17.14	62.86	93.75	50.00	31.25	6.25
Pachycheles rudis	Malacostraca	C	0.00	0.00	0.00	0.00	0.00	293.75
Pagurus sp.	Malacostraca	C	0.00	0.00	0.00	0.00	0.00	12.50
Pentidotea wosnesenskii	Malacostraca	N	17.14	0.00	0.00	0.00	0.00	12.50
Petrolisthes cinctipes	Malacostraca	N	992.86	314.29	781.25	750.00	93.75	2843.75
Anoplodactylus viridintestinalis	Pycnogonida	C	0.00	2.86	6.25	6.25	6.25	6.25
Pycnogonum stearnsi	Pycnogonida	C	0.00	0.00	0.00	0.00	18.75	12.50
Acorn barnacles (*Balanus glandula* & *Chthamalus dalli*)	Thecostraca	C	52857.14	25628.57	22393.75	67931.25	96362.50	89587.50
Pollicipes polymerus	Thecostraca	C	568.57	137.14	306.25	375.00	2912.50	2400.00
Semibalanus cariosus	Thecostraca	N	231.43	1.43	0.00	0.00	6.25	0.00
**Cnidaria**								
Anthopleura elegantissima	Anthozoa	C	24.29	12.86	43.75	93.75	100.00	50.00
**Echinodermata**								
Cucumaria pseudocurata	Holothuroidea	N	112.86	0.00	0.00	0.00	0.00	0.00
**Mollusca**								
Adula californiensis	Bivalvia	C	0.00	14.29	6.25	25.00	293.75	112.50
Leukoma staminea	Bivalvia	C	0.00	1.43	0.00	0.00	0.00	0.00
Modiolus carpenteri	Bivalvia	S	0.00	2.86	6.25	12.50	12.50	12.50
Mytilus californianus	Bivalvia	C	6645.71	20314.29	30968.75	34793.75	26275.00	17825.00
Amphissa versicolor	Gastropoda	C	0.00	0.00	0.00	0.00	0.00	12.50
Lacuna marmorata	Gastropoda	C	0.00	2.86	6.25	6.25	6.25	0.00
Littorina plena	Gastropoda	C	5.71	22.86	0.00	18.75	81.25	50.00
Lottia digitalis	Gastropoda	C	227.14	95.71	75.00	25.00	637.50	25.00
Lottia pelta	Gastropoda	C	1194.29	291.43	312.50	893.75	1968.75	3512.50
Lottia scabra	Gastropoda	S	5.71	50.00	56.25	12.50	18.75	0.00
Lottia sp.	Gastropoda	C	207.14	48.57	12.50	0.00	43.75	118.75
Nucella canaliculata	Gastropoda	N	28.57	5.71	12.50	18.75	93.75	500.00
Nucella ostrina	Gastropoda	N	120.00	42.86	50.00	206.25	112.50	1000.00
Tegula funebralis	Gastropoda	C	128.57	895.71	1087.50	2493.75	418.75	2250.00
Cyanoplax dentiens	Polyplacophora	C	11.43	110.00	31.25	6.25	18.75	0.00
Mopalia lionota	Polyplacophora	S	0.00	1.43	6.25	6.25	0.00	0.00
Mopalia muscosa	Polyplacophora	C	1.43	1.43	6.25	6.25	6.25	56.25
Nuttallina californica	Polyplacophora	C	0.00	1.43	0.00	0.00	6.25	0.00

Supplementary Figure S1. Depth of mussel bed for spatial comparison of four replicate plots. "F&H Subsample" is a subsample of the original plot sampled in 1941. 10–16 depth measurements were taken per plot before destructively sampling plots. Bars depict mean ± standard error. Shared letters above bars indicate means that did not differ (Tukey HSD, p>0.05).

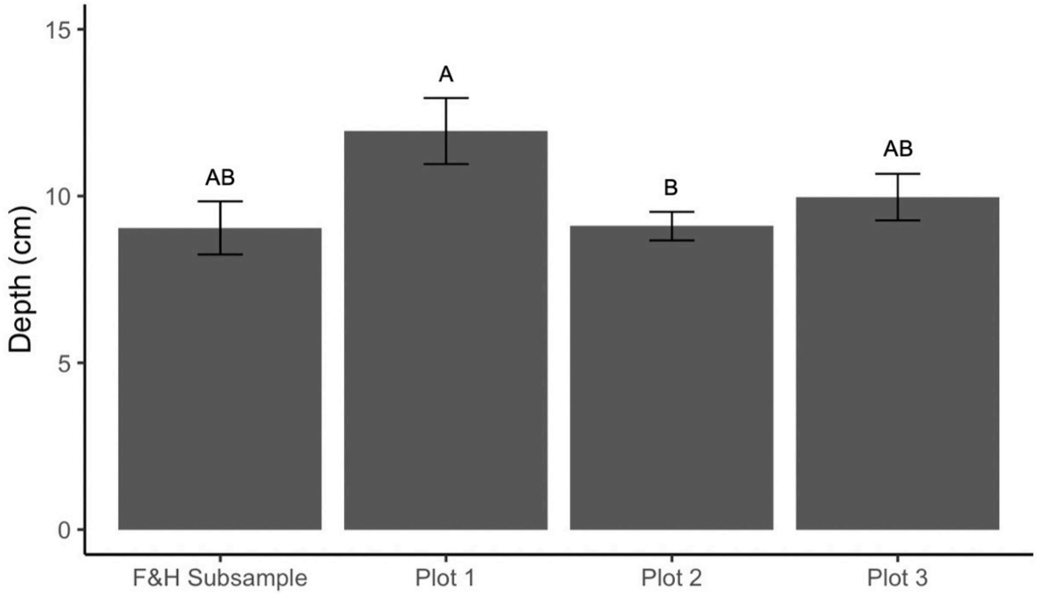

Supplementary Figure S2. Change in the log abundance of species within the mussel bed plot at Dillon Beach in 1941 versus 2019. (a) Variation in abundance among species colored by their Phylum. (b) Variation in abundance among species grouped by class within the arthropods and molluscs. Line is the one-to-one line. Points are slightly jittered to be able to see overlapping species. Taxonomic groups did not show consistent changes in abundance between timepoints.

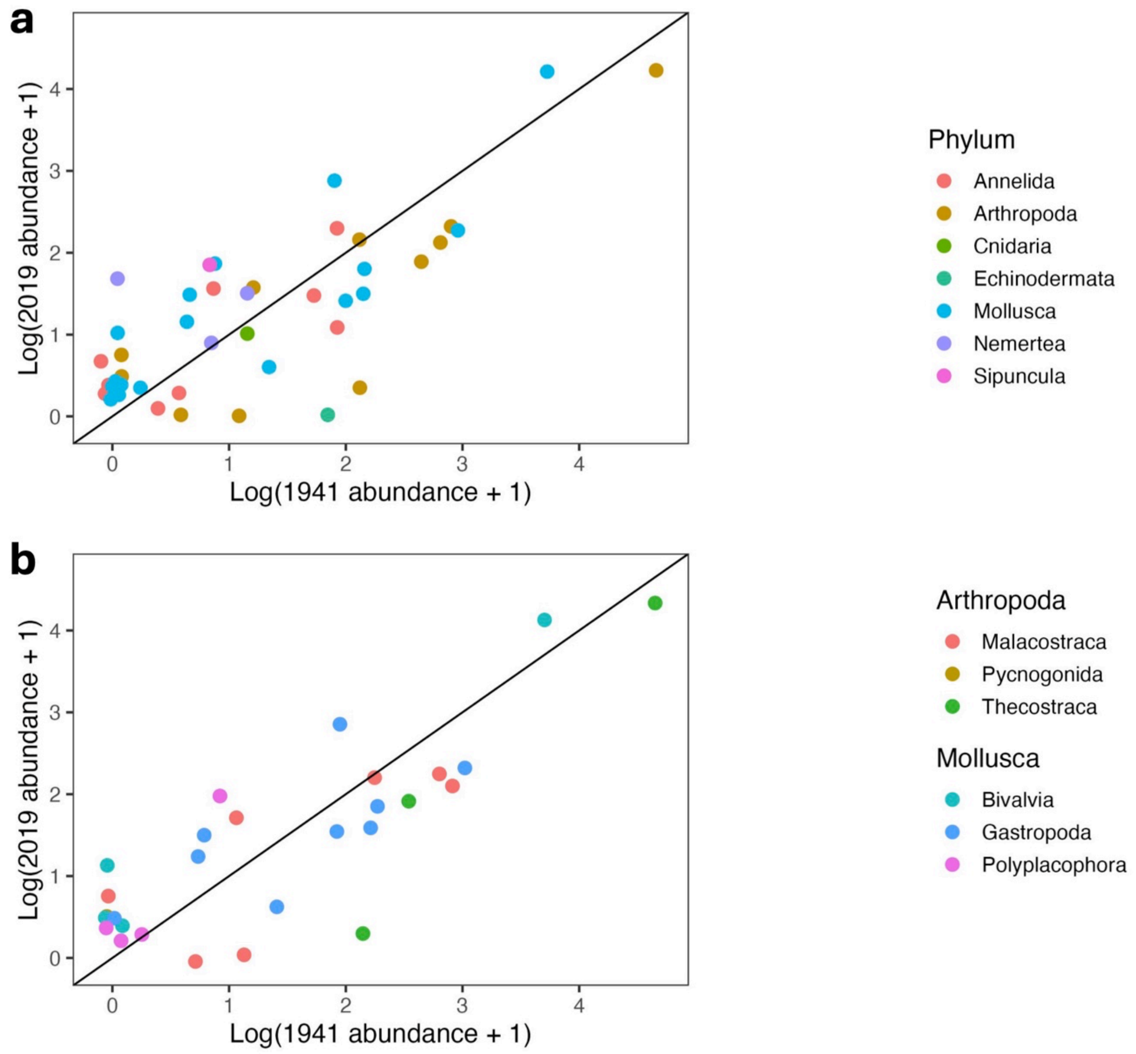

Supplementary Figure S3. Monthly seawater temperature from Bodega Head from 1956 to 2023. Monthly data were calculated from measurements taken at ~ 8:00am daily.

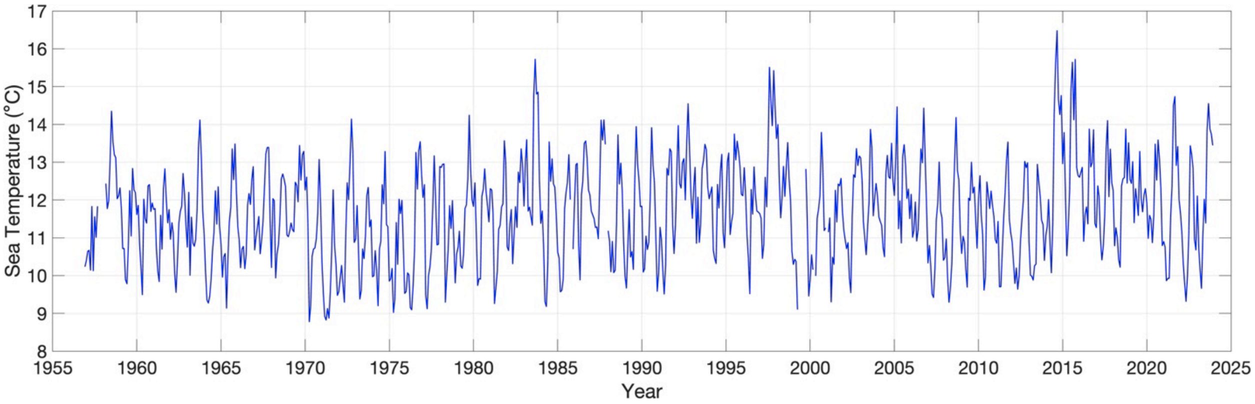

Supplement: Supplementary file 2 — Supplementary Material 2 [file 41598_2025_86105_MOESM2_ESM.pdf]
